# Supplementary material for: Are carbon emissions trading and green financial instruments synergistic? -Comprehensive quantitative research based on content analysis
Source: PLoS One. 2024 Mar 7;19(3):e0298601. doi: 10.1371/journal.pone.0298601 (PMC10919601; doi:10.1371/journal.pone.0298601)
Supplement: S1 Data — (ZIP) [file pone.0298601.s001.zip › Data/地级市Z_F工作报告.zip!/╝¬┴╓/┴╔╘┤╩╨/└┤╫╘╣1⁄2╓┌║┼í╛┬φ┐╦╩2╛▌═°í┐_2015.docx]

各位代表：

　　现在，我代表市政府，向大会作工作报告。请予审议，并请市政协各位委员提出意见。

　　一、2014年工作回顾

　　过去的一年，面对复杂的宏观经济形势和繁重的改革发展任务，市政府认真贯彻省委、省政府和市委的决策部署，保持战略定力，强化底线思维，勇于担当进取，奋力攻坚克难，辽源转型发展迈出新步伐，各项事业呈现新局面。

　　经济保持稳定增长。预计地区生产总值实现760亿元，增长6.5%，人均GDP突破10000美元；地方级财政收入完成26.8亿元，增长5.1%；社会消费品零售总额达到189亿元，增长13%；外贸进出口总额实现2.4亿美元，增长9%。按照新统计口径，固定资产投资增长17%；城镇、农村常住居民人均可支配收入分别增长3.7%和8.2%。节能减排等控制性指标全面完成。农业基础不断夯实。粮食产量达到47亿斤。新建现代农业园区和基地100个，蛋品、肉鸡、肉羊、梅花鹿、食用菌生产逐步实现规模化。金州生态产业园被列为省级产业开发区。东丰县域经济考评排名跃升至全省第九位。工业经济稳步增长。规上工业总产值实现1319亿元，增长10.5%；规上工业增加值超过400亿元，增长6.6%。产值超亿元企业达到213户，主导产业、特色产业趋稳回升。服务业发展持续向好。电子商务、信息服务、文化创意、物流配送、连锁经营等新型业态正在形成规模，旅游产业收入增长25%，第三产业增加值增长8.9%。经济结构进一步优化。三次产业比重由8.3：59.2：32.5调整到8.0：59.1：32.9，服务业增加值高于GDP增速2.4个百分点。民营经济活力充分释放。新登记私营企业1324户，增长15.2%；新登记个体工商户7216户，增长15%；主营业务收入突破1500亿元。新增个体工商户、民营经济增加值占GDP比重等指标居全省前列。

　　产业升级步伐加快。产业支撑体系构筑成型。装备制造和农产品深加工业占规上工业的57.3%；三个特色优势产业产值增长12.9%，其中高精铝加工、纺织袜业分别增长28.3%和14%；汽车零部件、矿山装备制造等产业集群发展势头强劲，新能源、软件等战略性新兴产业初具规模。经济发展后劲持续增强。实施亿元以上项目220个，其中新开工77个，利源精制深加工扩产、均胜汽车零部件、希玛生物DHA微藻等项目建成投产，麦达斯轻合金板带箔材、金翼循环经济产业园、美罗国际医药生产基地、汽车改装轨道客车自动涂装生产线等项目加快推进。科技创新步伐明显加快。启动申报国家可持续发展实验区，建立金州现代农业产业发展院士工作站，锂电池隔膜等9个项目列入省“双百计划”。新增吉林名牌产品13个，驰名商标和著名商标达到100个。吉林省化药中试中心和蛋品加工产业中试中心确定在我市建设。园区承载能力显著提升。辽源经济开发区完成扩容扩权，综合排名连续两年居全省省级开发区前5位，国家级开发区申报工作全面启动。各级各类园区基础设施趋于完善，产业特色更加鲜明，实现产值占全市工业比重接近70%。

　　城乡建设扎实推进。基础设施不断完善。伊开高速一期竣工通车，辽长铁路开工建设。杨木水库输水复线基本完工，东辽河、大梨树河城防及渭津河治理工程进展顺利。市区“八路一桥一广场”工程竣工，改造巷路22条，整修路基、硬覆盖30万平方米。改造各类地下管网197公里，新增供热能力300万平方米，城市内涝问题基本解决。城市管理日趋规范。国家级卫生城、园林城创建稳步推进，主城区全天候保洁，街路标牌全部更新，新增绿地面积26万平方米。拆除违章建筑2600余处，城市中心区全面实行“禁烧”，打击非法营运取得实效。智慧城市建设有序推进。启动云计算中心二次扩容，平安城市、智慧交通、智慧安防、智慧气象等平台投入运行。我市被列为全国信息惠民试点城市，荣获国家智慧城市建设成就奖。生态环境治理力度加大。东辽河流域治理、水源地保护工程有序推进。实现市属国有林场禁伐和集体林限伐。全面推进矿山湿地公园规划区企业搬迁和植被恢复。垃圾焚烧发电项目建成，垃圾填埋场投入使用，污水处理厂稳定达标运行。全民义务植树147.2万株。新增3个国家4A级景区。我市被确定为省级循环经济试点城市。重点镇建设迈出新步伐。省级示范镇金州乡生态农业、旅游产业初具规模，那丹伯、拉拉河、安恕等乡镇加速产城融合，横道河镇、渭津镇、建安镇被列为城乡建设全国重点镇。统筹推动新型城镇化与新农村建设，东丰县获得全国魅力新农村十佳县。

　　改革开放不断深化。重点领域改革全面启动。完成工商、质监、卫计委、食药监、城市规划管理等机构改革，政务服务中心和公共资源交易中心投入运行。减少行政审批项目180项，精简比例达到36.7%。工商注册资本认缴登记和“先照后证”制度全面推行。完善国有资产处置、预算信息公开、债务风险防控机制，实施市区分税制改革。创新助保池、土地收益保证贷款等投融资模式，组建榆银村镇银行。稳步实施农村土地确权登记颁证、统一城乡建设用地市场、农民变市民成本分担机制等城镇化配套改革。启动基础教育课堂教学改革，市属中学实现集团化发展。公立医院综合改革全面推开。文化、社会、生态文明体制改革有序推进。对外开放取得积极成效。引进域外资金520亿元，增长9.5%；实际利用外资2.8亿美元，增长12%。重点推动了与北京首农、北京合力清源、宁波华翔、吉林修正、上海创意产业协会等企业和机构的战略合作，签约落地浙江中农农副产品批发市场、阿里巴巴辽源产业带、长白山梅花鹿产业化基地等50个亿元以上项目，鑫晟汽车零部件、永建锅炉辅机配件等23个项目开工建设。中国华联总部落户辽源，成功进驻上海自贸区。第二届辽源国际袜交会取得丰硕成果。“激情冰雪、畅游辽源”冬季旅游隆重启幕。

　　民生状况进一步改善。完成49件惠民实事，当年民生投入达到50.6亿元。建设保障房32755套，回迁居民1.3万户，改造暖房子95万平方米，整治物业小区36个，托管弃管小区6个。创建省级高质量就业示范社区4个，新增城镇就业4万人，转移农村富余劳动力17.3万人，扶持成功创业5669人。再次提高养老金、失业金标准和医保报销比例，国企改制社保并轨试点企业失业金发放到位，在全省率先启动城镇居民大病医疗保险。城乡低保标准、机关事业单位住房公积金缴存比例达到全省中上游水平。临时救助困难家庭2.8万户，资助困难学生1.8万人次。新建农村标准化卫生室192个，实行基本药物制度的村级卫生室药价下降30%。新建养老机构9家，社区日间照料中心覆盖率达到97%。大力推动移风易俗，居民殡葬费用下降25%。市区新增停车泊位3000个，公交公营比例达到45.6%，龙嘉机场辽源城市候机楼投入使用。新增管道燃气用户1.2万户。解决1.68万农村人口饮水安全问题。城市24小时供水能力提高到90%。承办十七届省运会取得圆满成功。关东魁星文化庙会影响力显著提升。涌现出一批反映地域特色的优秀影视作品和文化产品，人民群众文化生活更加丰富多彩。

　　安全稳定局面持续巩固。城市社区全部实行网格化管理，立体化防控体系不断完善，覆盖城乡的视频监控系统一体化联网运行，刑事案件发案率下降10%，连续4年荣获全国见义勇为城市奖。建立信访大厅、市长信箱、市长公开电话协同办理反馈机制，强化隐患排查、矛盾化解、领导接访下访措施，信访结案率、积案化解率分别达到98%和94%。新的食品药品安全监管体系基本形成，餐饮服务、医疗器械、药品、保健品等领域专项整治取得实效。安全生产实现“五个全覆盖”，隐患整改率达到98.7%，各类安全事故下降35.7%。地方煤矿完成兼并重组，安全生产超过2000天。实现连续30年无重大森林火灾。积极推进国防教育和双拥共建，军政军民团结不断巩固。民族宗教、外事侨务、方志、档案、气象、地震、人防工作和妇女儿童、老龄、残疾人等各项事业协调推进。

　　政府自身建设不断加强。深入开展党的群众路线教育实践活动，严格贯彻中央八项规定精神，认真落实廉政建设各项要求，着力整治“四风”突出问题。会议文件、达标评比和各类节庆活动大幅减少，坚决停止新建楼堂馆所，清理超标准办公用房、公务用车取得阶段性成果，市级“三公”经费下降38.7%。加强法治政府建设，坚持科学民主决策，自觉接受人大、政协和社会各界监督。注重标本兼治，突出源头管控，大力整治发展软环境，从严查处违法违纪案件，政府公信力和执行力得到提升。

　　各位代表，过去的一年，我们在前所未有的挑战和困难中砥砺前行，成绩的取得来之不易。这是省委、省政府和市委正确领导、科学决策的结果，是市人大、市政协及社会各界有效监督、大力支持的结果，是全市人民奋发有为、团结拼搏的结果。在此，我代表市政府，向奋斗在各条战线上的广大干部群众，向历任老领导、离退休老同志，向各民主党派、工商联、无党派人士和人民团体，向驻辽部队、中省直单位及所有关心支持参与辽源发展建设的社会各界人士、海内外朋友致以衷心感谢和崇高敬意！

　　各位代表，面对新常态、新挑战，政府工作也遇到很多新矛盾、新问题。主要表现是：在巨大的经济下行压力下，部分行业产能过剩，企业开工严重不足，建筑、房地产业大幅下滑，对经济的负面影响加重；主导产业总体规模不大，特色产业支撑能力不强，新上项目规模偏小，在建大项目数量偏少，生产要素成本居高不下，融资瓶颈问题更加突出；财政收支矛盾特别是偿债压力不断增大，现有财力与发展和民生需求不协调、不匹配；政府职能转变还不到位，公职人员的担当意识、进取意识、实干精神、办事效率与群众期待存在一定差距。对此，我们将加大力度，创新举措，切实加以解决。

　　二、2015年工作安排

　　2015年，是“十二五”规划的收官之年，是全面深化改革的攻坚之年，也是全面依法治市的起步之年。市政府将深入贯彻党的十八届三中、四中全会和中央经济工作会议精神，全面落实省委十届四次全会、全省经济工作会议和市委六届四次全会作出的决策部署，主动适应经济发展新常态，牢牢把握稳中求进总基调，紧紧抓住东北振兴新机遇，继续实施投资拉动、项目带动、创新驱动战略，突出稳增长、调结构、促改革、惠民生等重点任务，更加注重转变发展方式，更加注重体制机制创新，更加注重强化法治思维，更加注重提高人民幸福指数，协调推进“五大建设”和“五大发展”，加快实现辽源转型发展新跨越。

　　主要预期目标是：地区生产总值增长7%左右；地方级财政收入增长6%以上；固定资产投资增长13%左右；社会消费品零售总额增长12%以上；城镇、农村常住居民人均可支配收入分别增长5%和8%左右；居民消费价格指数涨幅控制在3.5%左右；城镇登记失业率控制在3.9%以内；城镇化率提高1个百分点；节能减排确保完成省计划。

　　（一）保持经济平稳运行。始终把发展牢牢抓在手上，坚持扩大总量与提质增效并重，努力使经济增长处于合理区间。强化投资的关键作用。放大新一轮东北振兴和资源型城市转型等政策叠加效应，聚焦主导产业、基础设施、民生事业、科技创新、生态环保等重点领域，积极争取国家和省里更大支持。组织实施投资亿元以上项目200个，其中10亿元以上项目30个。着力推动麦达斯轻合金板带箔材、金翼循环经济产业园、方大锻造锻压生产线等项目投产达产，元隆达精密模具、东升冶金大型铸造、中农农副产品批发市场等项目开工建设。滚动实施重点企业技术改造，支持落地企业高效率、低成本扩能升级。扩大消费的基础作用。启动实施辽源市商业发展规划，加快网点布局和商圈建设，培育一批专业化大市场。鼓励引导居民改善住房条件，支持农民和流动人口在城市购房，切实减少二手房的流通环节和交易成本。增加汽车4S店和直销店数量，加快建设龙山汽车贸易产业园，进一步活跃二手车交易。完善生活服务、休闲娱乐、旅游、金融、地产品销售等电商平台，积极培育消费热点。推动农超农批农餐对接，加快发展农村快递业。举办具有地域特色的购物节、美食节、文化节、旅游节。提升出口的拉动作用。加强出口产品研发、品牌培育和公共服务平台建设，增加铝型材、鞋袜等产品出口份额，创建省级农产品出口基地。充分利用国家外贸政策和我省境外营销网络，开拓建筑塔机、选矿设备、特色农产品的海外市场。鼓励企业引进高端装备和专利技术，逐步实现以进促出、以进促产。发挥开发区的引擎作用。积极推动各类开发区和工业集中区提档升级，切实提高承载功能和投资强度，努力打造改革创新示范区、特色产业集聚区、转型升级先导区。完善辽源经济开发区建设总体规划，推进体制机制创新，打造优势产业集群，力争年内晋升国家级开发区。支持县域经济突破。加快发展立县产业，推动粮食、畜禽、果蔬等农牧业资源综合开发，提升钢铁、水泥、装备制造等县域工业支撑能力。坚持走内涵式发展道路，稳步推进新型城镇化建设，支持基础条件好的乡镇率先突破，推动重要节点镇差异化发展，打造一批产业强镇、人口大镇。加强经济运行协调。坚持分类指导、综合施策，帮助停产半停产企业恢复正常生产。加强产需衔接、互联互通，鼓励本地企业参与政府采购和政府主导项目采购。加快推进“气化辽源”工程，探索工业用电直供模式，切实降低企业用水、供热、物流等生产要素成本。

　　（二）调整优化产业结构。着眼于可持续发展，在重点领域和薄弱环节精准发力，加快打造辽源经济升级版。转变农业发展方式。发展精细种植业。建设30个高标准粮食高产核心区。推进食用菌、安石蟹稻等标准化、规模化生产。实施一批良种研发、农机推广和农田水利建设项目。发展精品养殖业。加快金翼蛋品、麒鸣肉羊、鹏翔肉鸡等现代牧业园区建设，新改扩建牧业小区30个，规模化养殖比重达到80%。加快推进无疫区建设。建立农畜产品质量安全监测中心。发展精深加工业。启动产业示范带动工程，加快现有农产品加工企业提档升级。依托特色资源和龙头企业，实施一批高附加值循环经济项目。重点抓好梅花鹿产品深度开发，大力开拓东丰、东辽绿色有机稻米的国内市场。壮大优势产业规模。加快传统产业转型升级，启动建设高精铝、汽车零部件等先进制造业园区，致力打造百亿级产业集群。突出提升、延伸、精制、品牌、整合的发展方向，推进利源、麦达斯等企业集团化发展，支持东北袜业调整产品结构，加速培育一批行业领军企业。积极盘活存量、优化增量，推进产能过剩行业结构调整和并购重组。大力推动城区老工业区、独立工矿区搬迁改造，启动国家级循环经济试点建设。优先发展现代服务业。培育设计研发、市场营销、投资咨询、技术信息、服务外包等专业组织，支持企业利用自有用地兴办自营性服务业，探索建立生产性服务业总部基地。实施旅游兴市战略，全面拓展观光旅游，突出发展休闲旅游，加快建设旅游基础设施和配套服务体系，积极推动鴜鹭湖生态休闲度假区、矿山湿地公园、扎兰芬围民俗文化园等旅游项目。加快推广电子商务、连锁经营、第三方物流等新型商业模式，做大阿里巴巴辽源产业带，引导更多企业加盟电商平台，促进线上线下融合发展。深入实施创新驱动战略。高度重视企业技术创新，鼓励建立技术研发机构，完善产学研用协同创新机制。全面推进国家可持续发展实验区建设，组织申报科技创新和科技成果转化项目30个以上。新建1个院士工作站、5个科技成果转化分中心，新增科技型企业10户以上。做大化药、针纺产业技术创新联盟，启动建设吉林省化药中试中心和蛋品加工产业中试中心，力争把东北袜业园打造成国家级科技企业孵化器。

　　（三）大力发展民营经济。把民营经济作为推进转型升级的主力军，在完善机制、破解难题上下大气力，让创业创新创造的活力充分涌动、持续迸发。不断壮大企业群体。积极推动万众创业、大众创新，完善中小企业服务体系，促进民间资本投资实体经济。实施小微企业培育计划，做实各类园区孵化器，新增小微企业400户以上。启动“小巨人”成长计划，支持中小企业扩能升级，实现小变大、弱变强、“四下”变“四上”。拓展政府购买公共服务领域，允许企业打捆承接政府订单，探索政府与社会资本合作的PPP模式。全面拓宽融资渠道。加强政银企合作，鼓励发展担保机构，充分发挥银行主渠道作用。切实降低贷款各环节财务成本，最大限度保证企业资金链安全。探索无形资产、企业信用、大额订单和现金流等新型融资方式，推进股权融资和融资租赁，鼓励企业在“新三板”上市。努力破解用工难题。积极推动校企联合、供需对接，扩大委托代培和订单培训，逐步解决熟练工短缺问题。采取网上招聘、驻校招聘、委托招聘等形式，吸引优秀大学毕业生来辽源创业就业。用好用活就业扶持基金，提高岗位技能培训的针对性和实效性。提升企业整体素质。引导企业在技术引进、产品研发、项目包装、市场开拓中注入品牌元素，打造和培育一批名牌产品和明星企业。加快民营企业股份制改造，完善法人治理结构，推动靠大联强、做大做强。积极营造企业家成长环境，让恪守诚信和履行社会责任的纳税人更有荣誉感、更有话语权，让更多优秀企业家脱颖而出。

　　（四）稳步推进城市建设。坚持前瞻性设计、高标准建设和精细化管理，加快建设宜居宜业生态智能的现代城市。完善城市建设规划。开展城市总体规划修编，完成城市风貌、市容市貌、城市路网、地下管网改造等专项规划。以打造现代服务业和都市工业集聚区为重点，统筹推动南部新城与经济开发区建设，加快形成城市商业副中心。坚持规划先行，创新融资模式，有序推动寿山新区和北部新城开发。加快基础设施建设。完成辽长铁路主体工程，启动伊开高速二期工程，抓好集双高速、四松电气化铁路建设前期工作。稳步推进东辽河、渭津河、大梨树河防洪工程。改造二次配水管网50公里、老旧供热管网30公里。实施市政“九路五带两园”工程，改造10条巷路和20条道路附属设施。适时启动人民大街畅通改造工程。提升城市管理水平。实施国家级卫生城、园林城创建攻坚战，深入开展市容环境综合整治，进一步提高城区环卫保洁标准，大力打击非法营运，全面清理占道经商和乱停乱放，实现“拆违”、“禁烧”常态化。推动市区烟花爆竹限放、禁放。确保天楹垃圾焚烧发电厂达标运行。推进智慧城市建设。重点实施政府公共信息平台、公共基础数据更新以及智慧社区、智慧医疗、智慧教育等应用项目，支持中天智慧城市产业发展集团和龙山科技智慧产业园建设。打破部门垄断，共享信息资源，探索可复制、可推广的城市管理和公共服务智能化模式，推进信息惠民、智慧城市建设试点变示范。

　　（五）全力开展招商引资。扩大对外交流与合作，启动大招商行动，为转型升级持续提供动力。引进更多重大项目。开展务实招商和精准招商，努力融入新一轮东北振兴战略布局和吉林中部城市群建设，将招商引资领域拓展到“一带一路”。借助第十届东博会、民企吉林行、国际采购商大会等载体和平台，吸引更多战略投资者在辽源布局产业、设置总部。重点推进与浙江中康、北京首农、宁波华翔、裕国国际、中欧商业协会、上海金融发展研究中心等企业和机构的合作。争取落地亿元以上项目60个，引进域外资金550亿元。坚持引资与引智并举，加速升级辽源的人才、产品和技术结构。加快推进签约项目。完善签约或开工项目跟踪落实机制，加快推进修正医药产业园、上海创意文化产业园、蓝天城儿童职业体验中心等项目开工建设。建立完善合同约束机制，着力解决制约项目落地的瓶颈问题，强力推动凯旋王国创意园、中意厨柜、辽河北岸城市综合体等项目加快建设。依法清理闲置土地，积极盘活各类要素，优先满足好项目、新项目建设需求。科学论证筛选甄别项目，建立优胜劣汰机制，提高招商引资质量。积极营造优越的政务、市场、法治环境，让来辽投资者落地生根、安心创业、放手发展。

　　（六）深入实施各项改革。着力推动重点领域改革，不断释放体制机制活力和改革红利，为转型升级创造更大空间。深化经济体制改革。进一步加大简政放权、放管结合改革力度，加快建立权力清单、责任清单和负面清单制度。继续压缩行政审批事项，推进“一站式”、“并联审批”和网上审批，实施行政审批再提速。深化城镇化配套改革。从提高城镇化质量和促进人的城镇化出发，推动扩权强镇、土地流转改革，建立统一的城乡建设用地市场和农村综合产权交易中心，创新城镇化投融资机制和农民变市民利益导向机制。深化财税体制改革。完善市区分税制改革，全面实施“营改增”，健全公共资源交易、债务管控、预算管理制度。开展政府投资项目绩效管理试点。深化安全生产领域改革。完善安全生产管理体系和责任体系，创新监管方式，建立多元化投入机制。加快推进金融创新。集聚一批金融机构和金融配套服务机构，支持民营企业发起设立民营银行。促进信贷资产证券化。鼓励发展互联网金融。有效防范金融风险。统筹推动社会事业改革。探索产教融合模式，加快职业教育改革创新。深入推进公立医院改革，促进公立、民营医疗机构协调发展。探索医养结合模式，争取我市列入省级养老事业和养老产业创新发展试点。

　　（七）加快建设幸福辽源。重点推进十项民生工程，以政府的“紧日子”换百姓的“好日子”。一是创业富民工程。扶持城乡创业3000人，新增城镇就业3.5万人。完善最低工资标准调整机制和职工工资正常增长机制。继续提高养老金、失业金标准，扩大民营企业参保范围。稳步推进厂办大集体改革。二是扶弱助困工程。配套实施低收入家庭医疗救助、临时救助、慈善救助、灾害救助和住房保障制度。建设残疾人康复中心。实现困难学生应助尽助。积极推进农村扶贫开发。三是住房保障工程。新建保障房6300套、37万平方米，回迁安置1万户。继续实施“暖房子”工程。切实推进已整治小区规范化管理，在弃管小区推行“准物业”自治模式。四是便民服务工程。深化公交企业改革，公交公营比例提高到60%。市区新增2000个停车泊位。城市24小时供水能力达到95%以上。倡导绿色生态殡葬方式，推动公益性墓地建设。五是满意教育工程。开展乱补课、乱办班专项整治，严查重处败坏师德师风行为。推进优秀教师资源集团交流和优质教育设施共享。严格执行招生政策，确保流程公开透明。六是健康医疗工程。建设标准化村卫生室147个，建立9个医疗服务联合体，推行分级诊疗模式。实施食品从农田到餐桌、药品全程可追溯监管，打击各类不法分子，保障人民生命健康。七是文化惠民工程。逐步开放市区学校体育活动场所，更新改造居民小区文体活动设施，开展徒步、骑行、广场舞等系列全民健身活动。推进公共文化资源共享，日军高级战俘营展览馆、矿工墓陈列馆向社会开放。八是信息惠民工程。发放一卡通市民卡30万张，实现基本公共服务平台缴费。推进教育、医疗、养老等领域的信息惠民工程，让城乡居民切实感受智慧城市建设带来的实惠和便利。九是暖心关爱工程。实施留守儿童、空巢老人关爱行动计划。扩大社会义工、慈善公益组织和志愿者群体。为一线环卫工人免费提供爱心早餐。让社会正能量温暖更多贫困家庭。十是幸福社区工程。加大对基层社区的人力、物力投入，赋予其承担职能和责任相匹配的财权、事权。开展文明社区、和谐邻里、幸福家庭创建活动，共同营造百姓心中的美好家园。

　　（八）切实保护生态环境。落实最严格环境保护制度，建设天蓝、地绿、水清的美丽辽源。大力防治水污染。严格执行重点流域污染防治规划，有效控制点源面源污染，加大仙人河整治力度，尽早实现东辽河出境断面水质达标。启动编制东辽河源头和杨木水库生态保护综合开发规划，努力解决水源地周边污染隐患，切实保障城市用水安全。全面治理大气污染。推进燃煤锅炉、餐饮业油烟、建筑扬尘、机动车尾气等污染防治。加强重点企业脱硫脱硝改造和国控污染源在线监控。市区实行PM2.5、PM10实时监测。制定实施秸秆综合利用规划，支持转化应用企业加快发展，统筹运用经济、行政、法治手段解决秸秆焚烧污染问题。加强矿山治理和土壤保护。深入推进矿山湿地公园建设，加快规划区域腾空搬迁和植被恢复，积极推动采煤沉陷区二次开发。坚决打击毁林开荒、蚕食林地行为，积极打造“公路绿廊”，启动省级森林城建设。改善农村人居环境。实施城乡结合部和近郊农村垃圾“村分类、镇收集、市处理”，推进垃圾无害化、减量化和资源化。着力整治农村环境，实施“六大工程”，加快建设一批美丽乡村。

　　（九）全面推进依法治市。依法全面履行政府职能，坚持法定职责必须为、法无授权不可为，坚决克服懒政怠政，严厉惩治失职渎职。加快建立科学完善的执法体系，全面落实行政执法责任制，严格规范自由裁量权，着力解决多头执法、重复执法、执法缺位等问题。依法保障人民群众合法权益，健全完善社会矛盾预警机制、利益诉求表达机制和救济救助机制，积极通过法律渠道化解信访突出矛盾。加快推进多层次、多领域依法治理，坚持严格规范公正文明执法，更加注重运用法治手段解决重点难点问题。依法推进平安辽源建设，重拳打击各类违法犯罪行为，努力营造安居乐业的社会环境。依法保障安全发展，强化安全检查和隐患整改，保证重点领域、重点行业、重点生产经营单位和人员密集场所安全，坚决防止较大以上事故发生。依法整治金融生态环境，严厉打击恶意逃债行为，不断提升企业诚信、社会诚信。依法推动国防建设，大力加强国防教育和双拥共建。依法加强民族宗教工作，进一步巩固民族团结、宗教和顺局面。深入开展“六五”普法，强化社会法治意识和法治信仰，努力使全体公民成为社会主义法治的忠实崇尚者、自觉遵守者和坚定捍卫者。

　　各位代表，绘就“十三五”美好蓝图意义非常重大，我们要站在时代前沿，立足辽源实际，以改革创新为动力，以人民幸福为目标，高质量编制“十三五”规划。特别要把构筑现代产业体系、推动经济转型升级作为工作主线，主动融入长吉图发展战略，全市上下奋战新五年，努力把辽源打造成全省中部率先转型成功、最具生机活力的重要支点城市和宜居宜业的新兴城市。

　　三、切实加强政府自身建设

　　不断巩固党的群众路线教育实践活动成果，深入推进作风建设和廉政建设，全力打造为民务实清廉的人民满意政府。

　　一是始终坚持为民执政。充分尊重人民主体地位，全部工作围绕实现群众意志和保障群众权益展开，带着感情和责任扎实做好利民惠民实事。以群众利益为导向推动改革发展，坚持问政于民、问需于民、问计于民，不断从人民群众中汲取智慧和力量，绝不违背群众意愿，绝不与民争利。进一步强化担当意识和进取精神，不推诿责任，不回避矛盾，不畏惧挑战，不瞻前顾后，始终保持开拓进取的锐气和昂扬向上的状态。

　　二是大力推进依法行政。坚决维护宪法法律权威，勇于自我革新，严格依法办事，切实做到有权必有责、用权受监督、失职要问责、违法要追究。把公众参与、专家论证、风险评估、合法性审查、集体讨论决定作为重大决策的法定程序，规范性文件、重要规划、重大事项必须经过充分协商和人大审议。加快转变领导方式，充分运用法治思维和法治方式深化改革、推动发展，化解矛盾、维护稳定。

　　三是更加注重务实理政。坚持从辽源实际出发，不耍嘴皮子、不摆花架子，抓铁有痕、踏石留印，及时消化旧矛盾、解决新问题。坚持把劲使在实事上，把钱花在刀刃上，坚决不搞华而不实的形象工程和劳民伤财的政绩工程。强力推进改革攻坚，敢开先河，敢涉险滩，敢过深水区，敢啃硬骨头。不断理顺政府职能，提高行政效能，全力打造全省行政审批最少、政务环境最优、服务效率最高地区。

　　四是坚决做到廉洁从政。深入贯彻落实中央八项规定精神，以铁的纪律和法之重器推进作风建设和反腐倡廉建设。强化重点领域、重要岗位、重大事权的监督和管控，真正把权力关进制度笼子。深化政务公开，推进“两个中心”规范运行，完善“三公”经费管理机制，确保公车改革等工作取得实效。健全惩治和预防腐败体系，积极主动、坚决果断地查处违法乱纪问题，始终保持干部清正、政府清廉、政治清明。

　　各位代表，辽源正站在新的历史起点上，无限光明的前景激励我们前行，艰巨繁重的任务需要我们担当。市政府要在市委的坚强领导下，主动适应、积极引领新常态，始终保持拼搏进取、奋发有为的精神状态，解放思想，坚定信心，务实重行，攻坚克难，紧紧依靠全市各族人民，奋力开创辽源转型发展新局面！
